# Does similarity in call structure or foraging ecology explain interspecific information transfer in wild *Myotis* bats?

**DOI:** 10.1007/s00265-017-2398-x

**Published:** 2017-10-29

**Authors:** Theresa Hügel, Vincent van Meir, Amanda Muñoz-Meneses, B.-Markus Clarin, Björn M. Siemers, Holger R. Goerlitz

**Affiliations:** 1 Sensory Ecology Group, Max Planck Institute for Ornithology, Seewiesen, Germany; 2 Acoustic and Functional Ecology Group, Max Planck Institute for Ornithology, Eberhard-Gwinner-Str. 11, Seewiesen, 82319 Germany; 3 Department of Animal Ecology and Tropical Biology (Zoology III), Julius Maximilian University of Würzburg, Würzburg, Germany; 4 Graduate School for Evolution, Ecology and Systematics, Ludwig Maximilian University of Munich, Planegg-Martinsried, Germany

**Keywords:** Acoustic tracking, Acoustic communication, Chiroptera, Eavesdropping, Heterospecific information transfer, Trajectory analysis

## Abstract

**Abstract:**

Animals can gain important information by attending to the signals and cues of other animals in their environment, with acoustic information playing a major role in many taxa. Echolocation call sequences of bats contain information about the identity and behaviour of the sender which is perceptible to close-by receivers. Increasing evidence supports the communicative function of echolocation within species, yet data about its role for interspecific information transfer is scarce. Here, we asked which information bats extract from heterospecific echolocation calls during foraging. In three linked playback experiments, we tested in the flight room and field if foraging *Myotis* bats approached the foraging call sequences of conspecifics and four heterospecifics that were similar in acoustic call structure only (acoustic similarity hypothesis), in foraging ecology only (foraging similarity hypothesis), both, or none. Compared to the natural prey capture rate of 1.3 buzzes per minute of bat activity, our playbacks of foraging sequences with 23–40 buzzes/min simulated foraging patches with significantly higher profitability. In the flight room, *M. capaccinii* only approached call sequences of conspecifics and of the heterospecific *M. daubentonii* with similar acoustics and foraging ecology. In the field, *M. capaccinii* and *M. daubentonii* only showed a weak positive response to those two species. Our results confirm information transfer across species boundaries and highlight the importance of context on the studied behaviour, but cannot resolve whether information transfer in trawling *Myotis* is based on acoustic similarity only or on a combination of similarity in acoustics and foraging ecology.

**Significance statement:**

Animals transfer information, both voluntarily and inadvertently, and within and across species boundaries. In echolocating bats, acoustic call structure and foraging ecology are linked, making echolocation calls a rich source of information about species identity, ecology and activity of the sender, which receivers might exploit to find profitable foraging grounds. We tested in three lab and field experiments if information transfer occurs between bat species and if bats obtain information about ecology from echolocation calls. *Myotis capaccinii/daubentonii* bats approached call playbacks, but only those from con- and heterospecifics with similar call structure and foraging ecology, confirming interspecific information transfer. Reactions differed between lab and field, emphasising situation-dependent differences in animal behaviour, the importance of field research, and the need for further studies on the underlying mechanism of information transfer and the relative contributions of acoustic and ecological similarity.

**Electronic supplementary material:**

The online version of this article (10.1007/s00265-017-2398-x) contains supplementary material, which is available to authorized users.

## Introduction

Animals need to gain information about their surroundings as the basis for their behaviours such as mating, foraging and predator avoidance (Dall et al. 2005). One rich source of environmental information is social information (Danchin et al. 2004), originating from con- and heterospecifics (Seppänen et al. 2007; Goodale and Kotagama 2008; Ruczyński et al. 2009; Dawson and Chittka 2012). Many animals, including insects, anurans, birds and mammals, use acoustic information for communication and species discrimination (Bradbury and Vehrencamp 2011). Acoustic information is independent of illumination and spreads well through the environment (Marten and Marler 1977; Marten et al. 1977; Wehner 1997; Dominy et al. 2001, 2004). Here, we investigated the role of a special kind of social and acoustic information, bat echolocation calls, for information transfer within and across species boundaries. While bird song evolved for communication between individuals, echolocation evolved for information acquisition by the calling individual (Schnitzler et al. 2003). Echolocation calls are thus adapted to optimise task-dependent echoes, and ecologically similar bats employ similar echolocation calls (Siemers and Schnitzler 2004; Jones and Teeling 2006). Although optimised for the needs of the caller, echolocation calls are also well audible to other individuals and reveal information about the location and current behaviour of the caller (Jones and Siemers 2011). Despite the general correlation between call structure and foraging ecology, deviations also exist. The echolocation calls of the European *Myotis* bats are mainly adapted to orientation close to background vegetation and do not necessarily reflect their differences in diet and foraging style (e.g. gleaning or aerial hawking). Likewise, the foraging ecologies of some species may partially overlap although their calls are clearly distinguishable (e.g. some *Myotis* and *Pipistrellus* species both hunting for swarming insects in riparian areas).

Intraspecifically, echolocation calls clearly have a communicative function (Jones and Siemers 2011). At least some bat species perceive conspecifics’ individual identity (Yovel et al. 2009), group membership (Boughman and Wilkinson 1998) and sex (Kazial and Masters 2004) from echolocation calls. In a foraging context, multiple bat species approach conspecific calls (Barclay 1982; Fenton 2003; Gillam 2007; Dechmann et al. 2009). This should be particularly beneficial for species foraging on ephemeral, patchily distributed and sharable resources such as insect swarms (Barta and Szép 1992; Beauchamp et al. 1997; Valone and Templeton 2002; Safi and Kerth 2007).

Interspecifically, however, the communicative function of echolocation is less understood. Echolocation calls may be used for species recognition, leading to reproductive isolation if calls change (Kingston and Rossiter 2004). Species discrimination based on echolocation calls was indeed shown for acoustically similar horseshoe bat species (Schuchmann and Siemers 2010). During foraging, some species appear to also approach heterospecific calls (Dorado-Correa et al. 2013; Übernickel et al. 2013). Open questions still are whether other bat species with different call structures are able to acoustically discriminate between sympatric heterospecifics, whether they use this ability to inform their behavioural decisions and space use, which acoustic information forms the sensory basis for this ability and whether acoustic information is combined with other prior information about the heterospecifics. The ability to discriminate between sympatric heterospecifics and to react with species-specific behaviours should be adaptive, for example by enabling a receiver to follow one heterospecific with similar foraging ecology to new or more profitable foraging sites, but not another one with dissimilar foraging ecology. In contrast to foraging bats, heterospecific information transfer has been clearly shown in other taxa and settings, such as during predator avoidance in fish based on olfactory cues (Wisenden et al. 1995) and in birds based on acoustic cues of other birds (Templeton and Greene 2007; Fallow and Magrath 2010) and mammals (Rainey et al. 2004), as well as based on visual information during foraging in bumblebees (i.e. Dawson and Chittka 2012) and habitat selection in birds (i.e. Parejo et al. 2004).

Here, we tested the basic question if bats use heterospecific information provided by echolocation calls emitted during foraging. We predicted that bats are attracted to calls of conspecifics and of heterospecifics with similar foraging ecology and tested two alternative hypotheses about the mechanism mediating attraction: (A) Attraction might be purely based on stimulus properties, with the bat evaluating acoustic similarity between the received call and its own calls (‘acoustic similarity hypothesis’; Balcombe and Fenton 1988; Übernickel et al. 2013). This should be adaptive given the general link between call structure and foraging ecology. (B) Alternatively, attraction might be based on a more complex process that combines stimulus properties and prior information about the caller to derive information about the caller’s foraging ecology (‘foraging similarity hypothesis’; Übernickel et al. 2013). Prior information might be innate or acquired by learning the call structure or species identity of other species present in the individual’s own foraging habitat.

In contrast to previous studies in echolocating bats, we designed a playback study to test both hypotheses simultaneously in our focal species, the long-fingered bat (*Myotis capaccinii*). Since *M. capaccinii* actively selects sites with high prey abundance (Almenar et al. 2009), we predicted that it should exhibit species-specific attraction to the foraging calls of other bats, depending on either the similarity in acoustic call structure or foraging ecology. We simulated high foraging activity of four co-occurring heterospecifics and of conspecifics as positive control. We selected the four heterospecifics based on their similarity in acoustic call structure and foraging ecology relative to the focal species, resulting in heterospecifics that were similar in acoustic call structure only (A+/F−), foraging ecology only (A−/F+), both (A+/F+) or neither (A−/F−). Acoustic similarity was based on spectro-temporal call characteristics. Similarity in foraging ecology was based on diet, foraging habitat and foraging style to identify species with access to the same food resources. Note, however, that sympatric species always differ to some degree to avoid competition (Schluter 2000), so we had to accept some variation in these traits (see the ‘Methods’ section for details). We conducted three playback experiments in the flight room and field. The first two experiments examined attraction to playbacks by individuals (flight room) and groups (field). The third experiment was conducted in the field and examined individual vocal behaviour and flight trajectories in response to playbacks. Specifically, we had two mutually exclusive predictions. Under the acoustic similarity hypothesis, we predicted that bats will approach acoustic simulations of foraging bats with similar acoustic call structure, independent of their foraging ecology. In contrast, under the foraging similarity hypothesis, we predicted that bats will approach acoustic simulations of foraging bats with similar foraging ecology, independent of acoustic call structure.

## Methods

### Species selection

**Fig. 1:**
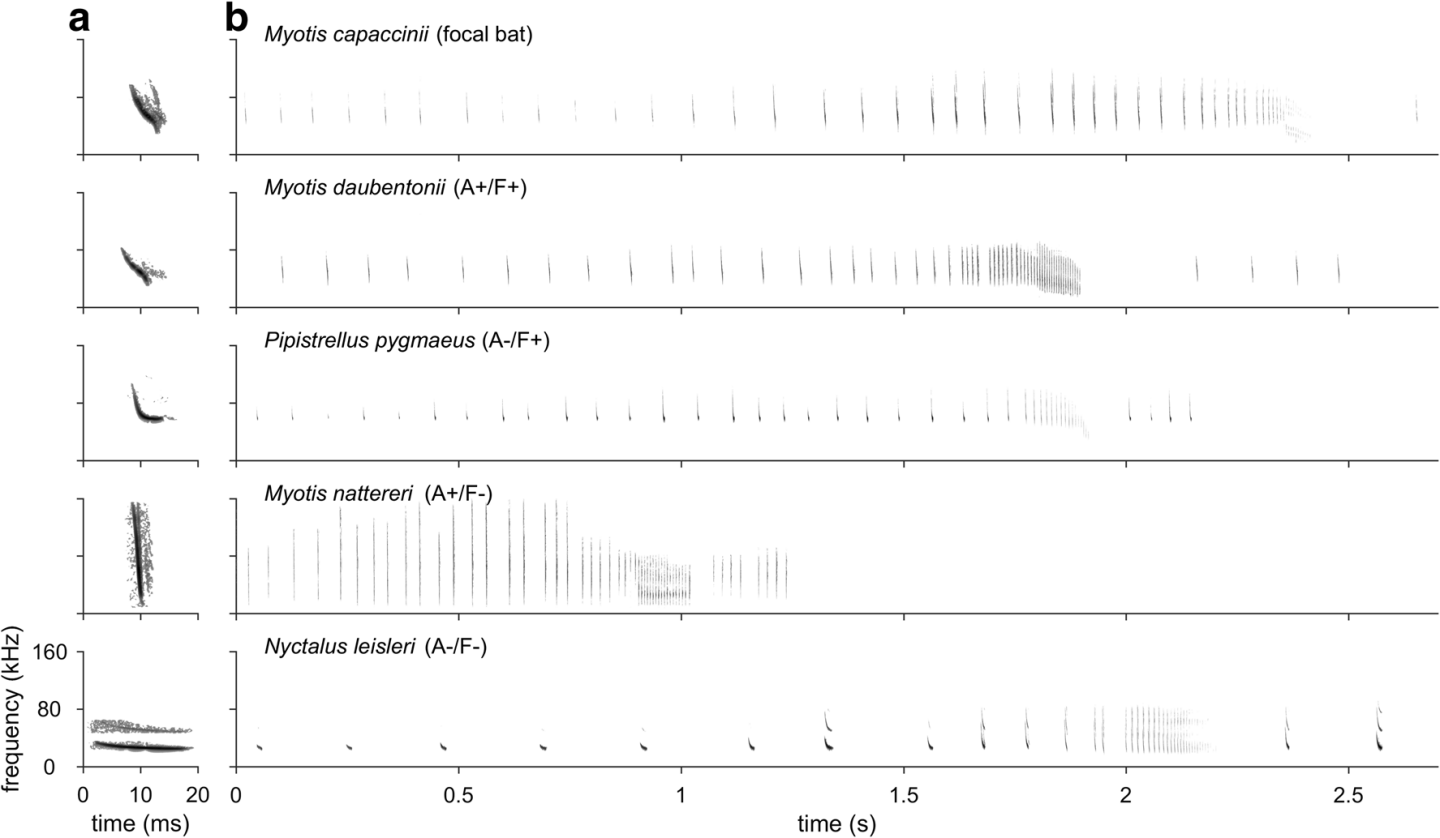
Example search call (**a**) and complete echolocation call sequence during foraging (**b**) of all five species used for playback

We generally predicted that bats are attracted to the echolocation calls of foraging bats if these calls indicate a resource that is profitable for and sharable by the eavesdropping individual (Barclay 1982; Dechmann et al. 2009; Dorado-Correa et al. 2013; Übernickel et al. 2013). Since resource profitability increases with increasing number and density of prey items (Stephens and Krebs 1986), it should be very high for many aquatic insects that occur in large numbers and dense swarms. We therefore planned our experiments for the focal bat species *Myotis capaccinii* that preys on insect swarms alone and in groups at many water bodies in Northern Bulgaria. Its diet mostly consists of flying dipterans with an aquatic larval phase (dominated by Chironomidae, yet also including other nematoceran insects, Brachycera, and other arthropods that are available in its habitat; Biscardi et al. 2007; Almenar et al. 2008). Its foraging style largely comprises of capturing prey from water surfaces (‘trawling’) and to a lesser extent in the air (‘aerial mode’; Kalko 1990; Spitzenberger and von Helversen 2001; Dietz et al. 2009). Correspondingly, its preferred foraging habitats are riparian areas with calm water sheltered by vegetation (e.g. Almenar et al. 2009). Its echolocation calls are downward frequency-modulated (FM) from 70–90 kHz down to 32–40 kHz with a duration of around 3–7 ms (Fig. 1, Fig. S1).

We chose four heterospecific bat species that are sympatric with *M. capaccinii* (Benda et al. 2003; Pandourski 2004; Popov et al. 2005) for our experiment (Table 1). They were classified into possessing similar/dissimilar acoustics and foraging ecology relative to *M. capaccinii* based on their acoustic call structure (also see Fig. 1 and Table 1 and references therein, Fig. S1) and their diet, foraging style and foraging habitat (Krapp 2001; Dietz et al. 2009; Denzinger and Schnitzler 2013); recognising however that there is a significant amount of biological and measuring variation and that no two species are ever identical.

- Acoustically similar, foraging similar (A+/F+): *Myotis daubentonii* is a trawling bat like our focal species. Its foraging niche largely overlaps with that of *M. capaccinii* (Biscardi et al. 2007), preying on a diversity of aquatic flying insects (Chironomidae, Ceratopogonidae and other nematoceran Diptera as well as Trichoptera; Vaughan 1997; Flavin et al. 2001; Swift and Racey 2009) over calm and sheltered water bodies, and, like *M. capaccinii*, also in the aerial mode (Jones and Rayner 1988; Bogdanowicz 1994; Dietz et al. 2009). Echolocation calls are of the same downward FM *Myotis*-type as in *M. capaccinii* with generally similar duration and start, peak and end frequency (Fig. S1), yet are somewhat more variable in frequency (Dietz et al. 2009) and reach lower end frequencies (Russo and Jones 2002).
- Acoustically dissimilar, foraging similar (A−/F+): *Pipistrellus pygmaeus* is an aerial-hawking bat foraging for insects close to vegetation edges, with a strong preference for riparian habitats (Bartonička and Řehák 2004; Davidson-Watts et al. 2006). Its diet includes a high percentage of nematoceran insects (Chironomidae, Ceratopgonidae), Trichoptera, Brachycera and further small dipteran insects (Barlow 1997; Bartonička et al. 2008). Although *P. pygmaeus* is not a trawling bat, we classified it as similar in foraging ecology due to this spatial and dietary overlap; its calls should thus indicate a suitable foraging habitat to *M. capaccinii*. Its search calls are of the FM/QCF type, starting with a variable downward FM part around 80 ± 15 kHz and ending with a quasi-constant-frequency (QCF) part around 55 kHz (± 5 kHz) and thus clearly differ from the typical broadband FM *Myotis*-type. Call duration is up to 10 ms and thus longer than in *M. capaccinii*.
- Acoustically similar, foraging dissimilar (A+/F−): *Myotis nattereri* is an active gleaning bat that echolocates arthropods hanging close to or sitting on top of vegetation, mostly within forest habitats (Siemers and Schnitzler 2000; Dietz et al. 2009). Its diet contains mainly diurnal Diptera which are gleaned from their nightly resting places (Vaughan 1997). Its foraging ecology thus clearly differs from trawling bats, requiring high manoeuvrability close to background structures and analysing prey echoes in highly cluttered acoustic environments. Its calls are of the typical downward FM *Myotis*-type (Fig. 1). We therefore classified it as acoustically similar to *M. capaccinii*, noting however that its calls are more broadband and shorter than those of *M. capaccinii* and *M. daubentonii* as an adaptation to its dense foraging habitat (Fig. 1, Fig. S1) and thus more distinguishable from those two species than those two species can be distinguished from each other (e.g. Russo and Jones 2002).
- Acoustically dissimilar, foraging dissimilar (A−/F−): *Nyctalus leisleri* is a typical forest bat that forages by relatively fast and unmanoeuvrable flight in the edge and open space of forests, meadows and aquatic habitats (Vaughan et al. 1997; Waters et al. 1999; Kaňuch et al. 2007) where it catches prey in the aerial mode (Dietz et al. 2009). Its diet often overlaps with that of *M. capaccinii* by including nematoceran Diptera including some Chironomidae, although it is more variable and also contains larger insects such as Brachycera, Coleoptera and Lepidoptera (Rydell et al. 1995; Vaughan 1997; Shiel et al. 1998), and can even be dominated by Lepidoptera (Kaňuch et al. 2005). Despite the partial dietary overlap, we classified it as having dissimilar foraging ecology due to the mostly different foraging habitat and style. Its search calls are of the FM/QCF type and are longer (up to 20 ms) and lower in frequency (ranging from about 40–60 kHz down to 21–26 kHz) than those of *M. capaccinii* and are thus clearly different and distinguishable.

**Table 1:**
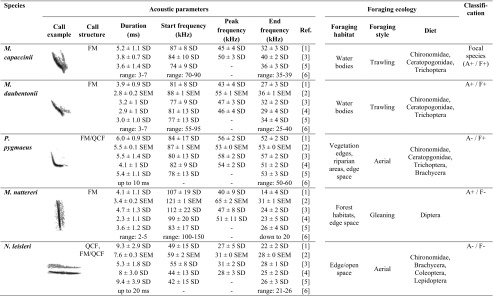
Literature values for acoustic call parameters and foraging ecology of the five bat species used in this study, and their classification as similar/dissimilar relative to the focal species *M. capaccinii*. Also, see Fig. 1 for exemplary call spectrograms

Start frequency: frequency at the start of the call = highest call frequency. Peak frequency: frequency of maximum energy. End frequency: frequency at the end of the call = lowest call frequency. Note that exact definitions and methods for measurements may differ between studies. Classification: indicates similarity (+) and dissimilarity (−) in acoustic call structure (A) and foraging ecology (F) relative to the focal species *M. capaccinii*. To reduce potential bias due to different call analysis methods, we only used studies that compared echolocation calls of multiple European bat species:

[1] Obrist et al. 2004

[2] Parsons and Jones 2000

[3] Russo and Jones 2002

[4] Vaughan et al. 1997

[5] Walters et al. 2012

[6] Dietz et al., 2009

*FM* frequency-modulated, i.e. call frequency changes over time; *QCF* quasi-constant-frequency, i.e. call frequency does not change much over time (i.e. is quasi-constant). A FM/QCF call starts with a frequency-modulated part and ends with an almost constant-frequency part

The selection of our focal species was further supported by species sociality. Only species that forage in groups can benefit from interspecific information transfer, and benefits should be highest for species foraging on sharable and ephemeral resources. Indeed, those species with a high proportion of ephemeral insects also form male groups more often, including our focal species *M. capaccinii* (and *M. daubentonii*, see next paragraph for focal species in the field; Safi and Kerth 2007). This confirms that our focal species exploits sharable resources, and supports our assumption that they should benefit from interindividual information transfer.

Using playbacks of these four heterospecific bats, we can test our two hypotheses based on their mutually exclusive predictions: The acoustic similarity hypothesis predicts that *M. capaccinii* approaches playbacks of the acoustically similar heterospecifics *M. daubentonii* and *M. nattereri*. In contrast, the foraging similarity hypothesis predicts that *M. capaccinii* approaches playbacks of the heterospecifics with similar foraging ecology, *M. daubentonii* and *P. pygmaeus*. *N. leisleri* served as a negative control and should not be attractive. The flight room experiment was conducted with *M. capaccinii* that were caught in caves and held in short-term captivity. The two field experiments were conducted with wild free-flying bats. Since acoustic differentiation between many *Myotis* species is difficult, we did not differentiate between recordings of *M. capaccinii* and *M. daubentonii* in the field experiments, which were both foraging at our field study sites in variable proportions (estimated to range from 0.2:0.8 to 0.8:0.2 based on previous captures at these sites, A. Hubancheva, personal communication). The field recordings are thus a combination of both species (but exclude recordings of other species), meaning that the presented playbacks of *M. capaccinii* and *M. daubentonii* were either a con- or heterospecific playback to the recorded bat. However, since we classified both species as ecologically and acoustically similar, they can replace each other as the positive control, without affecting the predictions for the other playback stimuli.

### Echolocation call sequences used as stimuli

**Fig. 2:**
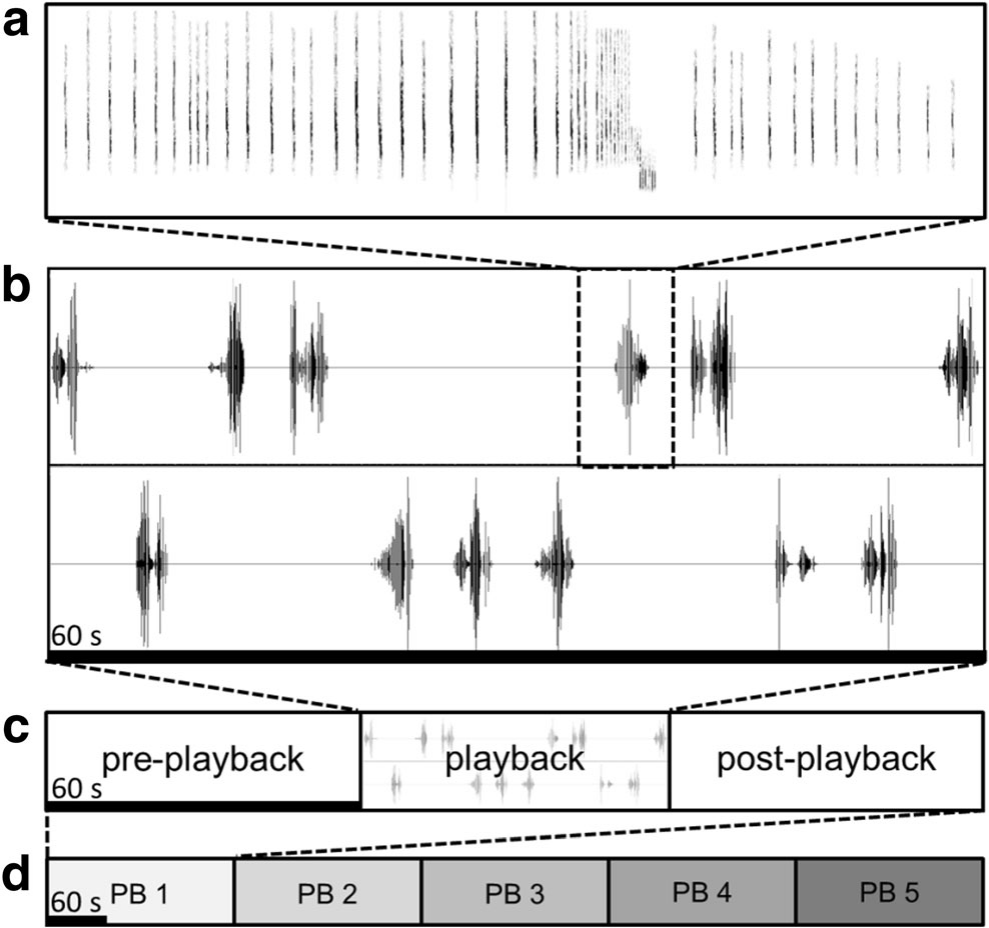
Playback stimuli and sequence for Exp. 1 and 2. Twelve echolocation call sequences recorded during foraging (**a**) were combined into 60-s-long stereo playback files (**b**). One full trial for each playback species consisted of three experimental phases (pre-playback, playback and post-playback phase; **c**). Trials of five playback species were presented in block-randomised sequence (**d**)

As playback stimuli, we used complete echolocation call sequences recorded during foraging that included search, approach and final (feeding buzz) phases (Fig. 1), sometimes with subsequent search calls after the feeding buzz. The acoustic structure of search calls differs between bat species and thus provides species-specific information about species identity, habitat and (foraging) ecology. In contrast, feeding buzzes differ less between species, yet are emitted when attacking prey and thus convey information about the behavioural context (foraging) and prey availability (number of emitted feeding buzzes). In contrast to previous studies, we therefore specifically refrained from presenting either search calls or buzz calls only. We used 13–55 different call sequences per species (see Table S2 for recording details). Sequences had a median duration of 2.39 s (interquartile range: 2.13–2.68 s, min: 0.58 s, max: 3.22 s; Table S1). Sequences were high-pass filtered at 10 kHz (8th order butterworth-filter) to reduce low-frequency background noise, filtered with the speakers’ inverse frequency response to ensure a flat frequency response and faded in/out over the first/last 10% of their duration. The digital peak amplitude of the loudest call per sequence was set to 90% full scale and played at the maximum amplification without clipping, resulting in playback levels of 118 ± 2 dB peSPL re. 20 μPa (114 ± 2 dB SPL RMS; mean ± SD; *N* = 10 analysed calls) for *N. leisleri* and 111 ± 2 dB peSPL (104 ± 3 dB SPL RMS) for the other four playback species at 10 cm in front of the loudspeaker. Differences between species were caused by overcompensation of the lower frequency range, as confirmed by later recalibration measurements. Overall, our playback levels were about 9–15 dB fainter than natural source levels (Holderied and von Helversen 2003; Surlykke et al. 2009; Goerlitz et al. 2010), yet still high and well audible to bats, resulting in naturally varying species-specific detection ranges of about 19 (*M. nat.*), 22–26 (*M. cap.*, *M. dau.*, *P. pyg.*) and 67 (*N. lei.*) m for a conservative bat hearing threshold of 20 dB peSPL (calculated for our median weather conditions of 19 °C and 90% relative humidity and peak frequencies of 65, 52, 50, 56 and 27 kHz, respectively). Like real bats, the loudspeakers were directional, thus roughly halving the detection ranges for bats at 40–90° off-axis to the loudspeakers’ main axis. Foraging call sequences were either played individually (Exp. 3) or as a series of 12 foraging sequences of one species combined into a single playback file for a total duration of 60 s (Exp. 1 and 2, see below and Fig. 2).

### Assessment of the playback stimuli as indicators of profitable foraging patches

To assess the quality of the foraging patches simulated by our playbacks, we analysed the naturally occurring bat activity and prey capture rate at our field sites and compared them to our simulated activity and capture rate. We first counted the naturally occurring number of bat passes and feeding buzzes emitted by *M. capaccinii/daubentonii* during all 1-min-long pre-silence phases (*N* = 499) of Experiment 2 (for details, see below). We also measured the duration of the first bat pass of *M. capaccinii/daubentonii* in 100 randomly selected pre-playback phases. To obtain capture rate, we normalised the feeding buzz counts to (i) the total observation time (=total recording duration), (ii) the number of bat passes and (iii) the total duration of bat activity (=number of bat passes multiplied by pass duration).

### Individual flight activity in response to echolocation calls (Exp. 1)

To test both hypotheses under controlled conditions where the bats’ behaviour was not influenced by unknown factors such as prey availability or competition, we measured individual flight activity relative to playback type in a flight room split into two compartments.

#### Animal capture and husbandry

Twenty-seven *Myotis capaccinii* (15 males, 12 females; > 1 year, post-lactating) were caught with mist or hand nets from two colonies within 100 km from the Siemers Bat Research Station in Tabachka, Northern Bulgaria. Bats were kept for 4–29 days in cages with maximally 8 individuals under naturalistic conditions (light on 8 am–9 pm, 26 °C average temperature, 67% average relative humidity) and free access to water. Food (mealworms) was provided during training on the pools in the flight room, or after training by hand if bats did not gain sufficient weight.

#### Setup

Experiments were conducted in an echo-attenuated flight room (4 m × 8 m × 2.4 m, L × W × H), which was separated into two equally sized compartments (4 m × 4 m) by a curtain, leaving an opening of 0.7 m at one end to allow the bats to change compartments (Fig. S2). Each compartment was equipped with a water pool (1 m × 2 m) for foraging. Two loudspeakers (Vifa, Avisoft Bioacoustics, Glienicke, Germany) per compartment were mounted on the wall opposite to the opening at a height of 1.7 m to play echolocation call sequences via a soundcard (USG Player 216H, Avisoft) and the Avisoft RECORDER software from a laptop computer. Four synchronised cameras (WAT-902H ultimate, Watec Co., Ltd., Japan; 1.4/4.5 mm wide-angle lens, SV-03514, VS Technology, Japan; Digi-Protect Video Surveillance Software, ABUS, Wetter, Germany) monitored the complete flight room under near-infrared illumination to record bat behaviour as well as an IR-LED that lit up during sound playback for synchronisation. The bat was always visible on at least one camera. Playback and data acquisition were controlled from an observation room outside the flight room.

#### Stimuli

Per individual tested, we prepared five unique 1-min-long playback sequences (one sequence per playback species). Each playback sequence was a two-channel sound file (one channel per speaker in a compartment), containing 12 foraging echolocation call sequences randomly chosen from the species’ pool of foraging call sequences, which were equally distributed in time and pseudo-randomly (Gellermann 1933) across channels, with six sequences per channel (Fig. 2a, b). Each playback sequence was only presented once.

#### Protocol

Before data acquisition, bats were allowed to fly for 2–19 nights in the flight room for acclimatisation, either alone for 20 min or in groups for 2–7 h. Equal amounts of mealworms were placed on both pools, and acclimatisation ended when bats ate mealworms from both pools for at least two successive nights. Then, the experiment was conducted the following night, without offering food on the pools. After releasing a single bat into the flight room, we presented playbacks of all five playback species in an order blockwise randomised across individuals and video recorded the flight activity in both compartments. Each of the five trials per individual consisted of three 1-min-long experimental phases (pre-playback, playback and post-playback phase; Fig. 2c, d). One of the playback stimuli described in the previous section was presented during the playback phase of each trial, either in the left or right compartment (pseudo-randomised, Gellermann 1933). If the bat was flying at the end of the post-playback phase, the pre-playback phase of the next trial was started immediately; otherwise, we waited until the bat took flight again. During the pre-playback phase, a trial was aborted if the bat flew for less than 20 s, and restarted from the beginning as soon as the bat took flight again.

#### Analysis

Due to initial technical issues during data acquisition, the first seven animals were excluded from the dataset. For the remaining 20 individuals (8 males, 12 females), we manually measured the total time the bat spent flying in each compartment per experimental phase by watching the video recordings blind to experimental phase and playback species. We excluded 21 experimental phases, in which the individuals flew for less than 20 s, resulting in a final dataset of flight activity during 279 experimental phases of 20 bats, with *N* = 14–20 for a given experimental phase and playback species. We tested the effect of playback species on the proportion of time the bats spent flying within the playback compartment (=flight time in playback compartment divided by total flight time) using linear mixed models (R version 3.3.2, package: lme4 Bates et al. 2015). The full model included proportional flight time (dependent variable) as a function of playback species, experimental phase and their interaction (fixed factors) and individual number (random factor). Comparison with a reduced model without the interaction showed a significant effect of the interaction (see the ‘Results’ section). We thus calculated separate models for each playback species, including proportional flight time as a function of experimental phase (fixed factor) and individual number (random factor). We tested for an effect of experimental phase on the proportional flight time by comparing the full model to the reduced model without phase, using likelihood ratio tests and AIC comparison. To compare phases, we performed post hoc Tukey tests for multiple comparisons (package: multcomp; Hothorn et al. 2008).

### Total group flight activity in response to echolocation calls in the field (Exp. 2)

To test both hypotheses under naturalistic foraging conditions, we repeated the above experimental design in the field by recording the acoustic activity of trawling *Myotis* bats (*M. capaccinii* and *M. daubentonii*) during foraging while presenting the same 3-min-long experimental trials.

#### Study area

Experiments were conducted during five evenings in July/August 2013 and 15 evenings in July/August 2015, between 8:15 pm to 23:58 pm, at five water bodies (lakes at Basarbovo, Bazan, Krasen and Svalenik; Cherni Lom River at Tabachka; Northern Bulgaria) used by *Myotis* bats as foraging grounds. Locations were tested in blockwise randomised order, with four recording nights per location.

#### Setup and protocol

Two speakers (Vifa, powered by Avisoft Player 216H, Avisoft Bioacoustics) and a microphone (CM16/CMPA, recorded onto USG 416H (2013) or USG 116HM (2015), Avisoft Bioacoustics) were set up 0.7–2.7 m above the water surface. The microphone faced horizontally towards the centre of the water body (perpendicular to the shore line), with the speakers directly below facing 45° to its left and right. Per night, we presented 25 3-min playback trials (each consisting of 1-min pre-playback, playback and post-playback phases) in blocks of five trials (one trial per playback species per block), and simultaneously recorded bat echolocation, using Avisoft RECORDER software (Avisoft Bioacoustics). In total, we thus presented 500 trials, with 100 trials per playback species (20 trials per playback species and site). One trial (*M. capaccinii* at Cherni Lom River in 2015) was excluded due to technical failure. Bat activity was estimated per each 1-min phase by counting the number of bat passes in the call recordings, blind to phase and playback species. Bat passes were counted by viewing time windows of 0.5–1 s in the spectrogram overview display in SASLab Pro (Avisoft Bioacoustics; 20 kHz high-pass filter; 256 FFT, Hamming window, frame of 100%, threshold of 30). We defined a bat pass as consisting of at least three consecutive search calls, which were separated by less than three times the mean call interval of the previous three calls (Übernickel et al. 2013).

#### Analysis

We tested the effect of playback species on the number of bat passes (dependent variable) using generalised linear mixed models (Poisson family), with playback species, experimental phase and their interaction as fixed factors, and trial number and location as random factors to account for our repeated measures design (R version 3.3.2, package: lme4 Bates et al. 2015). Comparison of this full model with a reduced model without the interaction term showed a significant effect of the interaction (see the ‘Results’ section). We thus calculated separate models for each playback species, including number of passes as a function of experimental phase, trial number and location. We tested for an effect of the experimental phase on the number of passes by comparing the full models to the reduced models without the experimental phase, using likelihood ratio tests and AIC comparison. To compare phases, we performed post hoc Tukey tests for multiple comparisons (package: multcomp; Hothorn et al. 2008).

### Individual flight reactions to bat calls in the field (Exp. 3)

To analyse individual reactions to echolocation calls in the field, we conducted another playback experiment and recorded bat echolocation calls on a four-microphone array to analyse vocal behaviour as well as three-dimensional flight trajectories of foraging *M. capaccinii* and *M. daubentonii*.

#### Study sites, setup and protocol

Experiments were conducted at six evenings between 8:50 pm and 0:22 am in July and August 2013 at three sites with foraging *M. capaccinii* and *daubentonii* bats along the river Cherni Lom in the vicinity of Tabachka. We recorded bat calls with a planar symmetrical star-shaped four-microphone array positioned close to the rivers’ shore (CM16/CMPA microphones, USG 416H soundcard, Avisoft RECORDER software, Avisoft Bioacoustics; 60 cm distance between the central and the peripheral microphones). Playbacks were presented via a loudspeaker (Scanspeak powered by a USG player 216H, Avisoft Bioacoustics) placed at the same height in 5-m distance to the array’s central microphone, at 45° to the left or right in front of the array. As playbacks, we used the individual foraging sequences instead of the combined 1-min-long playback sequences used in the previous two experiments (Fig. 1). The order of the five playback species plus a silence control was blockwise randomised for each evening, with the specific playback file per playback species randomly chosen from the pool of available foraging sequences, and variable number of repeats per evening until bat activity ceased. A playback was started manually when bat calls were visible on the onscreen display of the microphone signal. Recorded calls were saved to hard drive from 5 s before onset of the playback (via a ring buffer) until several seconds after the end of the playback when no calls were visible anymore.

#### Trajectory reconstruction and selection

We reconstructed three-dimensional flight trajectories based on the time-of-arrival-differences of their echolocation calls between the peripheral and the central microphones, using custom written scripts for Matlab (The Mathworks Inc., Natick, MA, USA), and manually viewed and checked all flight trajectories. We then excluded all flight paths that had less than six 3D-positions, were shorter than 1 s, ended earlier than 1 s after the start of the playback and started later than 1 s after the end of the playback. This resulted in a total of 128 trajectories (14–37 per playback type, 13–80 per site) from an unknown number of individuals, with durations from 1–2.9 s and 6–22 3D-positions. If echolocation calls were not detected automatically, their timing was manually marked and added to the trajectory for analysing the bat’s vocal behaviour.

#### Analysis

Each trajectory was interpolated in 10-ms steps using cubic smoothing splines (Matlab function csaps, intermediate smoothing). The difference between the raw and smoothed positions was minimal (median 2–5 cm, Q3 < 10 cm, corresponding to < 1% difference normalised to distance, confirming a very low-noise localisation; Fig. S3). We calculated six parameters for each trajectory: relative flight height (m), distance to loudspeaker (m), instantaneous curvature (1/m), instantaneous change in flight direction (°/s), tortuosity per 500 ms bins and overall tortuosity of the total trajectory. If bats approached the loudspeaker in response to one of the playback species, we expected to find reduced distance to loudspeaker and increased values for the total range of flight height and the other four trajectory parameters related to trajectory curviness. Furthermore, we hypothesised that bats might also react by changing their temporal call pattern in one of two different ways. Either, they might call less (=increased call interval) to listen for the simulated bat calls, or they might call more (=reduced call interval) to increase their own sensory information flow about a close-by bat.

We performed two separate analysis approaches to test for those playback-specific changes: First, we extracted for each trajectory the extreme values of these eight parameters (total range of relative flight height, minimum distance to speaker, maximum values of the four other trajectory parameters related to curviness, and minimum and maximum call interval) and compared them between playback species (see below). Second, we compared the four temporally finely resolved trajectory parameters (flight height, distance to loudspeaker, curvature, change in flight direction) and the call interval over time to test for playback-species-specific temporal changes. We hypothesised that reactions might occur either directly after playback onset or after buzz onset. We therefore used the time when the first call of the playback and the first call of the buzz arrived at the bat’s position as two reference time points for the temporal analysis of each trajectory. Per trajectory, we calculated the mean trajectory parameters in 0.5-s-wide bins relative to both reference time points (bin edges = …−1, −0.5, 0, +0.5, +1 s re. reference time point) and then compared the trajectory parameters in the bin before the reference time point (−0.5–0 s) to the second bin after the reference time point (+0.5 – +1 s). As reference time points for the silence control (which had no playback and thus no reference time points of its own), we used the mean values of both reference time points of all trajectories with *M. capaccinii* playback. Since trajectory parameters are correlated, we first applied a principal component analysis to the z-scored trajectory parameters for both analysis approaches (six and four trajectory parameters for the extreme value and the temporal analysis, respectively). We then calculated the Euclidean distances between all trajectories in the space of the first three principal components (PC). Since PC-scores and Euclidean distances were partially non-normally distributed (estimated with QQ-plots), we used randomisation tests for testing the effects of playback species and time.

For the extreme value analysis, PC1-PC3 scores explained 60.7, 16.8 and 11.8% of the variation in the trajectory parameters. We used a non-parametric permutation-based one-way MANOVA (PERMANOVA, Fathom-toolbox for Matlab, Jones 2015) to test whether the PC-score Euclidean distance were greater between playback species than within playback species, and one-way ANOVAs with randomization tests (Matlab) to test for an effect of playback species on the minimum and maximum call intervals. The temporal analysis was conducted twice, once for each reference time point. We used non-parametric permutation-based two-way MANOVAs (PERMANOVA, Fathom-toolbox) to test for effects of playback species, time and their interaction on the Euclidean distance matrix. PC1–PC3 scores explained 47.0, 25.6 and 23.2% of the variation for playback-start as reference time, and 50.5, 25.5 and 22.6% for buzz-start as reference time. If required, this was followed by subsequent one-way-PERMANOVAs per playback species to test for species-specific effects of time. We used two-way ANOVAs with randomization tests (Matlab) to test for effects of playback species, time and their interaction on call intervals.

## Results

### Assessment of the playback stimuli as indicators of profitable foraging patches

We counted 3331 passes and 738 feeding buzzes of *M. capaccinii/daubentonii*, distributed across 443 and 223 1-min-long pre-silence phases, respectively (out of a total of 499). Both distributions, the number of bat passes and particularly the number of feeding buzzes per minute of observation were right-skewed with median values of 7 bat passes and 1 feeding buzz per minute of observation (Fig. S4A, B). Normalised to the number of bat passes and their median duration (2.26 s, Fig. S4D), this resulted in a natural median capture rate of 0.05 buzzes per bat pass (Fig. S4C) and 1.3 buzzes per minute of bat activity (Fig. S4E). In contrast, our experiments simulated 12 bat passes per minute (~ 2× more than observed naturally) and 12 feeding buzzes per minute (~ 12×), resulting in a capture rate of 1 feeding buzz per bat pass (~ 20×; Fig. S4A-C) and 23–40 feeding buzzes per minute of bat activity (Fig. S4E; depending on the species-specific median playback duration of 1.51–2.59 s, Table S1, Fig. S4D for *M capaccinii/daubentonii*). In summary, the presented feeding buzz rate was an order of magnitude higher than the natural rate at the experimental sites, no matter whether analysing feeding buzzes per minute of total time, per bat pass or per minute of bat activity. This suggests that our playbacks simulated highly profitable foraging patches which should be attractive to other bats within earshot.

### Individual flight activity in response to echolocation calls (Exp. 1)

**Fig. 3:**
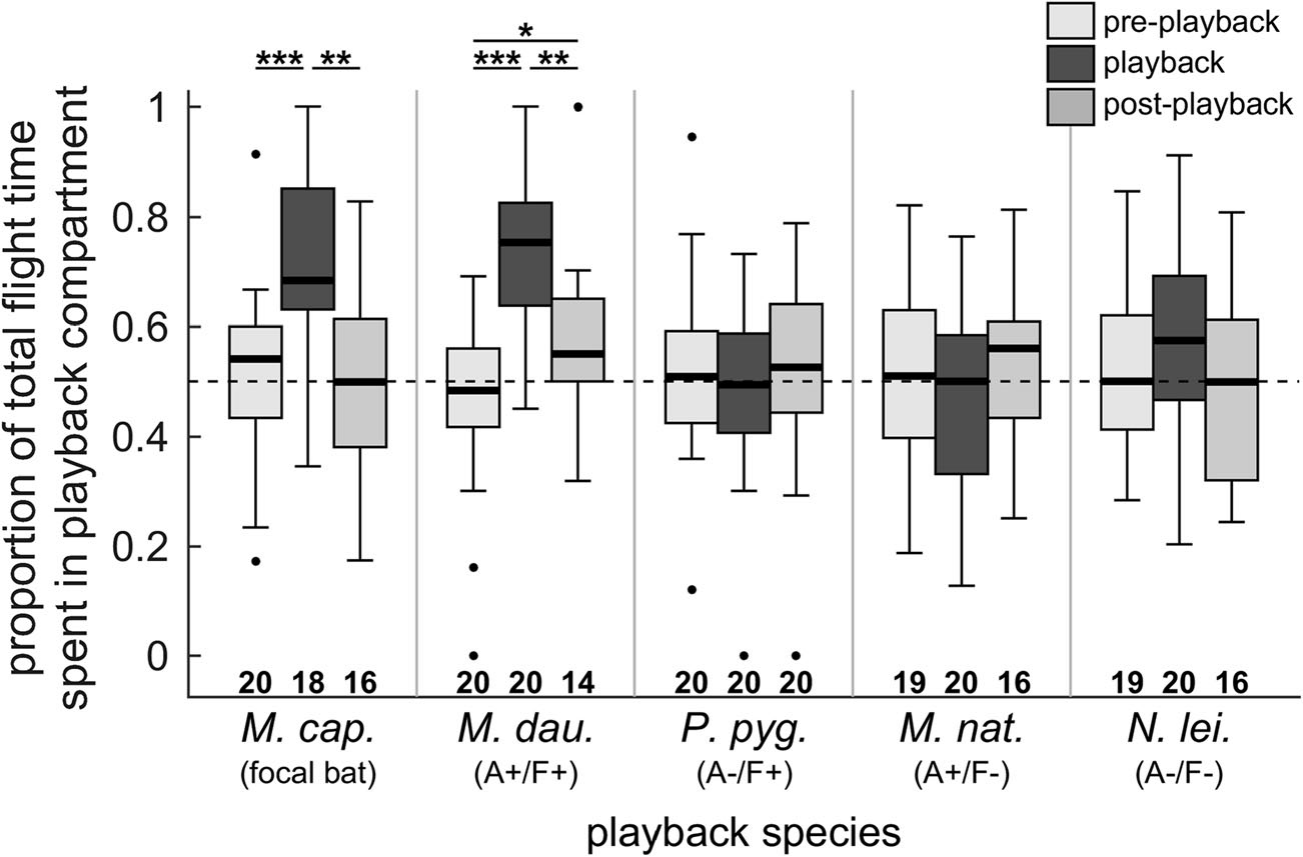
Proportion of total flight time spent in the playback compartment by individual *M. capaccinii* bats, as a function of playback species and experimental phase (Exp. 1). Echolocation call sequences of five different playback species were presented during the playback phase. Box plots present median, quartiles, whiskers at up to 1.5 times the interquartile range beyond the quartiles, and outliers. Asterisks indicate significant differences between experimental phases (Tukey post hoc test). A+/− and F+/− indicate our classification as similar/dissimilar in acoustic call structure (A) and foraging ecology (F) to the focal species. Horizontal dashed line indicates equal proportions of time (0.5) spent flying in both compartments

We presented foraging call playbacks of five bat species to individual *M. capaccinii* flying in a flight room, and measured the proportion of time spent flying in the compartment with playback. Median total flight duration was 56 s (quartiles 43–60 s) per experimental phase, which did not vary significantly over the course of a trial for any of the five playback species (Kruskal-Wallis test, unadjusted *p* = 0.065–0.656, i.e. non-significant already without correction for multiple testing). The minimum adequate model included the interaction between the fixed factors experimental phase and playback species (log-likelihood test to the model without interaction: *p* < 0.001, AIC −190.10 vs. −169.67). Thus, the effect of the experimental phase on the flight time depended on the playback species. Separate models per playback species showed a significant effect of the experimental phase on the proportion of time spent flying in the playback compartment for playbacks of *M. capaccinii* and *M. daubentonii* (log-likelihood test, *p* < 0.001 for both, AIC −28.203 vs. −16.568 and −42.71 vs. −19.48 respectively), but not for any of the other three playback species (Fig. 3). When presenting *M. capaccinii* and *M. daubentonii* playbacks, bats spent significantly more time in the playback compartment than during the silent pre- and post-playback phases (Tukey post hoc tests, *p* < 0.001 for both; Fig. 3). Proportional time in the playback compartment was larger by +0.21 (confidence interval 0.07–0.34) and +0.21 (CI 0.07–0.35) for *M. capaccinii* playback, and by +0.27 (CI 0.17–0.38) and +0.15 (CI 0.04–0.27) for *M. daubentonii* playback compared to the silent pre- and post-playback phases, respectively. In summary, bats were attracted by the calls of their conspecifics and of the heterospecific species with similar call design and foraging ecology.

### Total group flight activity in response to echolocation calls in the field (Exp. 2)

**Fig. 4:**
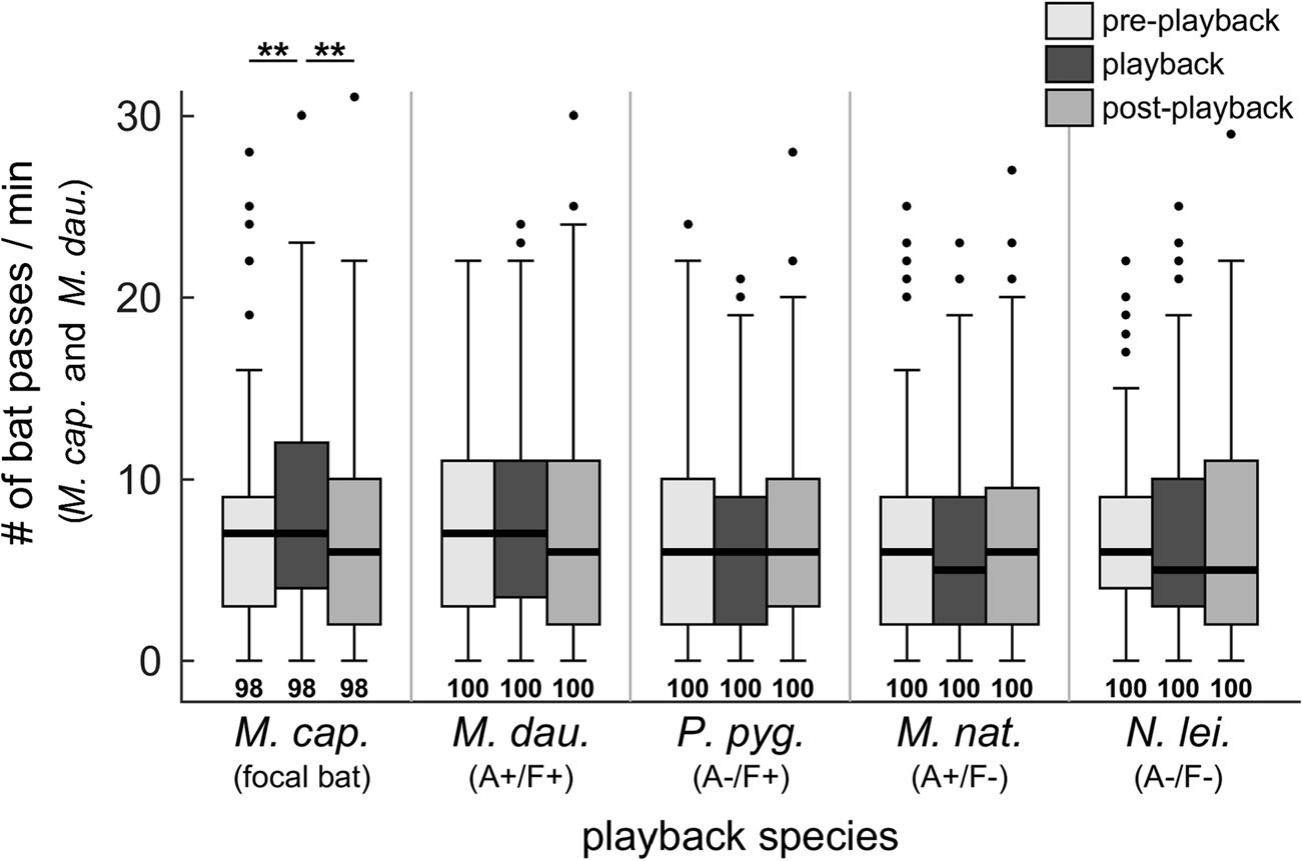
Group activity of *M. capaccinii* and *M. daubentonii* in the field measured as number of passes, as a function of playback species and experimental phase (Exp. 2). Echolocation call sequences of five different playback species were presented during the playback phase. Box plots present median, quartiles, whiskers at up to 1.5 times the interquartile range beyond the quartiles, and outliers. Asterisks indicate significant differences between experimental phases (Tukey post hoc test). A+/− and F+/− indicate our classification as similar/dissimilar in acoustic call structure (A) and foraging ecology (F) to the focal species

We investigated the response of bats to echolocation calls on the group level in the field at five foraging sites by counting bat passes. It is likely that both *M. capaccinii* and *M. daubentonii* were foraging at all sites. Since their calls look similar and are difficult to discriminate with certainty, we did not discriminate between them and present their combined reaction to the playbacks, but excluded the passes of any other recorded species. This does not affect our predictions since *M. capaccinii* and *M. daubentonii* were classified as acoustically and ecologically similar and can replace each other as the positive control. Number of *M. capaccinii* and *M. daubentonii* bat passes recorded per experimental phase ranged from 0 to 31 with an overall median of 6 (Fig. 4). The minimum adequate model included the interaction between the fixed factors experimental phase and playback species (log-likelihood test to the model without interaction: *p* = 0.012, AIC 8112.7 vs. 8114.9). Thus, the effect of the experimental phase on the number of passes dependend on playback species. Separate models per playback species showed a significant effect of the experimental phase on the number of bat passes for *M. capaccinii* playbacks (log-likelihood test, *p* < 0.001, AIC 1627.9 vs. 1638.3), a trend for *M. daubentonii* playbacks (log-likelihood test, *p* = 0.081, AIC 1751.2 vs. 1752.2) and no effect for the remaining three playback species (Fig. 4). During playback of *M. capaccinii* call sequences, the number of bat passes during the playback phase was significantly larger than during the silent pre- and post-playback phases (Tukey post hoc test, *p* = 0.008 and < 0.001, respectively; Fig. 4), although with very small effect sizes of +0.15 (CI 0.03–0.27) and +0.18 (CI 0.06–0.31) bat passes only, respectively. As in the flight room, bats were attracted to conspecific calls and showed a positive trend towards the heterospecific species with similar call design and foraging ecology.

### Individual flight reactions to bat calls in the field (Exp. 3)

**Fig. 5:**
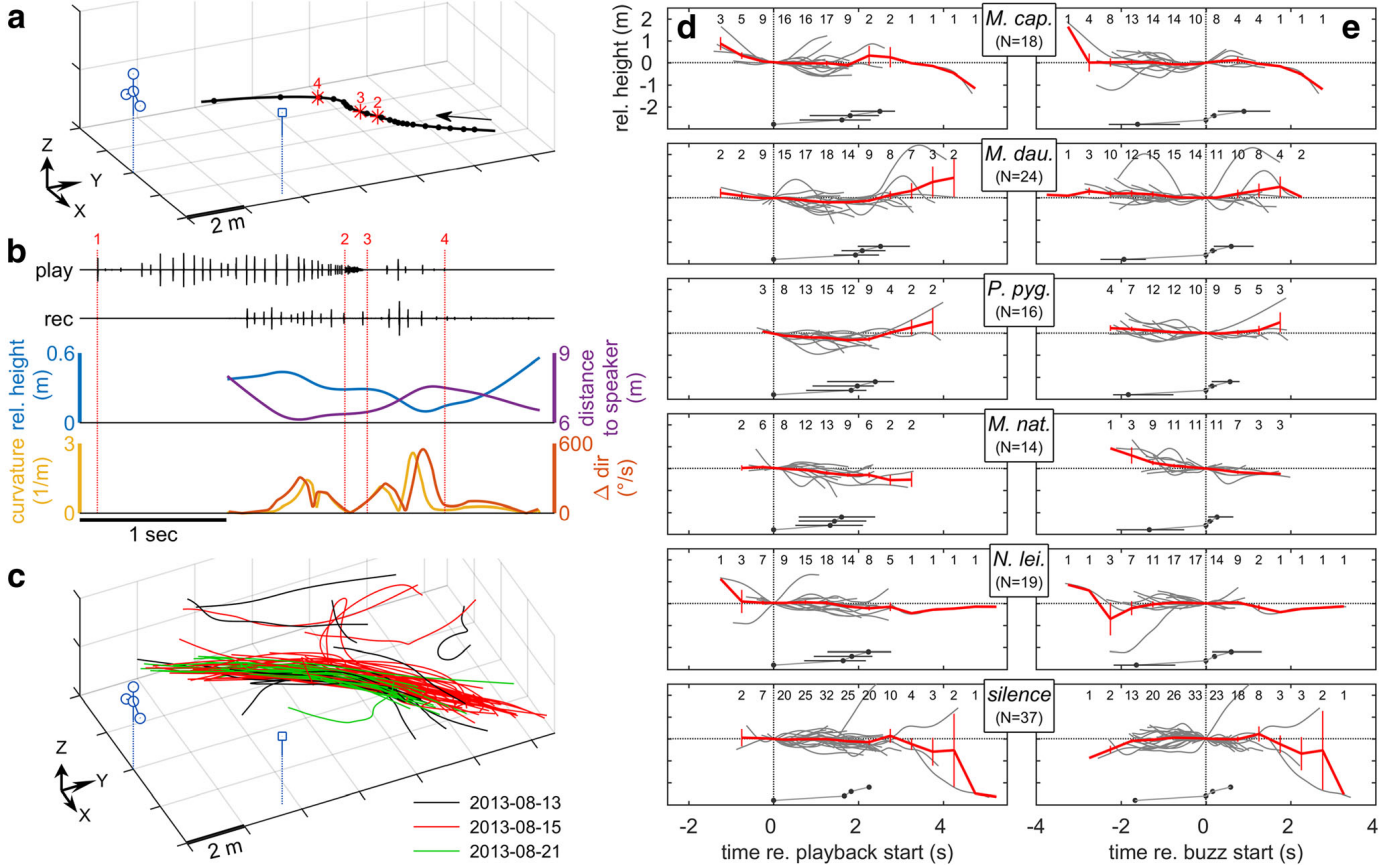
Trajectories and flight height of *M. capaccinii* and *M. daubentonii* in the field (Exp. 3). **a** Exemplary flight trajectory of one bat in response to *M. daubentonii* playback. Arrow shows flight direction; dots indicate bat positions at call emission; asterisks indicate bat positions at exemplary reception times of the playback (start/end of playback/buzz). Microphone array and playback loudspeaker are shown in blue, with solid lines indicating their tripods and dotted lines visualising their position on the XY-plane. **b** *M. daubentonii* playback, call recording and four trajectory parameters of the trajectory shown in **a**. Vertical dotted lines mark the start/end of the playback sequence and the feeding buzz, respectively; small numbers correspond to the numbers in **a**. If bats showed a goal-directed approach towards the playback, we expected to see increased flight height (since the loudspeaker was positioned above the bats), reduced distance to the speaker and larger curvature and changes in flight direction. **c** Flight trajectories (*N* = 80) recorded over three nights (colour-coded) at one river foraging site. **d + e** Relative trajectory height (*N* = 128) as a function of playback species and time relative to the playback, with *t* = 0 set as the start of the playback (**d**) and start of the buzz (**e**), respectively. Lines are individual data (grey) and means ± SEM binned per 0.5 s (red). Small numbers at the top indicate number of trajectories per bin. Dots with horizontal bars below the data indicate the mean playback time and the min/max-range across all playbacks for the four reference times indicated by numbers 1–4 in **a** and **b**: start/end of the total playback sequence and of the feeding buzz

We analysed 128 call sequence recordings and the corresponding three-dimensional flight trajectories of flying *M. capaccinii* and *M. daubentonii* bats in the wild (Fig. 5a, c) to investigate their individual short-term vocal and flight behaviour in reaction to foraging call sequences of five different bat species. If bats reacted to and approached the playback loudspeaker, we predicted increasing range of flight height (since the loudspeaker was positioned above the bats average flight height), decreasing distance to the speaker and increasing trajectory parameters related to trajectory curviness (curvature, change in flight direction, tortuosity; Fig. 5b, d, e). In addition, we predicted that bats change their calling behaviour by either calling less (=increased call interval, to listen for the simulated bat calls) or more (=reduced call interval, to increase information flow about a close-by object; Fig. 5b). Trajectory height (Fig. 5d, e), curvature (Fig. S5B) and direction change (Fig. S5C) did not obviously vary over time. In contrast, distance to speaker generally decreased over time (Fig. S5A); however, it is confounded by the bat’s general flight along the river which also affects its distance even without reacting to a playback. Like the trajectory parameters, call interval did not clearly decrease or increase after the start of the playback or the feeding buzz (Fig. S5E). We first compared the extreme values of the trajectory parameters and call interval of each trajectory across playback species. In contrast to our predictions, we found no significant differences between playback species, neither for the PC1-PC3 scores of the extreme values of the trajectory parameters (one-way-PERMANOVA, F(5122) = 0.767, *p* = 0.659; Fig. S6A-F), nor the minimum and maximum call interval (one-way-ANOVA with randomization test, *p* = 0.191 and 0.300, respectively; Fig. S6G). Second, we tested for changes in the same trajectory and vocal parameters over time, comparing the bin before to the second bin after one of two reference time points (start of the playback and start of the feeding buzz, respectively; Fig. 5d, e, Fig. S5). When referenced to the start of the playback, we found a significant effect of time (two-way-PERMANOVA, *F*(1128) = 10.5, *p* = 0.001), but not playback species (*F*(5128) = 0.872, *p* = 0.619) and their interaction (*F*(5128) = 0.844, *p* = 0.600) on the PC1–PC3-scores. Despite the lacking interaction, we tested each playback species separately and found a significant effect of time only for *M. daubentonii* playback (Bonferroni-Holm adjusted *p* = 0.006), but not for the other playback treatments (p_adj = 0.08–1.058). When referenced to the start of the feeding buzz, neither species (two-way PERMANOVA, *F*(5131) = 1.00, *p* = 0.419) nor time (*F*(1131) = 0.640, *p* = 0.586), but their interaction (*F*(5131) = 2.42, *p* = 0.015) significantly influenced the PC1–PC3 scores. Subsequent separate tests per playback species found no significant effect of time for any of the playback species (p_adj = 0.096–1.182). Call interval was not significantly influenced by playback species, time and their interaction, for both reference time points (two-way ANOVAs with randomization tests, *p* = 0.108–0.645; Fig. S5D).

## Discussion

We used echolocating bats to test the acoustic similarity against the foraging similarity hypothesis in a combined experimental design in the flight room and field. Many echolocating bats are attracted to the calls of other bats (e.g. Barclay 1982; Übernickel et al. 2013), which provide social information about profitable foraging patches (Templeton and Giraldeau 1996; Coolen et al. 2003; Dawson and Chittka 2012). In the flight room, individual *M. capaccinii* were only attracted to foraging conspecifics and to the acoustically and ecologically very similar heterospecific *M. daubentonii*. In contrast, foraging *M. capaccinii* and *M. daubentonii* in the field were largely not attracted to any of the playbacks, except for slight tendencies to approach *M. capaccinii* and *M. daubentonii* calls, indicated by increased group activity (Fig. 4) and altered individual flight trajectories (Exp. 3). In contrast to our predictions, bats did not approach the species only similar in acoustic call structure (A+/F−, *M. nattereri*) or foraging ecology (A−/F+, *P. pygmaeus*) more than expected by chance. Based on our initial predictions, our results thus support neither the acoustic similarity hypothesis nor the foraging similarity hypothesis, which might be explained by several reasons.

Classifying the similarity of co-occurring species has an inherent problem since co-occurring species need to differ to avoid competition (Schluter 2000). The foraging ecology of *M. capaccinii* is clearly more similar to *M. daubentonii* (A+/F+) than to *P. pygmaeus* (A−/F+), despite existing overlap in diet, foraging style and foraging habitat between *M. capaccinii* and *P. pygmaeus*. Likewise, the calls of *M. capaccinii* and *M. daubentonii* are more similar to each other than the calls of *M. capaccinii* and *M. nattereri* (A+/F−). This might have resulted in the species we designated as acoustically (*M. nattereri*) and ecologically (*P. pygmaeus*) similar to *M. capaccinii* not being similar enough for a positive reaction to occur.

Bats are auditory specialists and might be capable of perceiving small differences in acoustic stimuli. Constant-frequency bats, like horseshoe bats, possess extremely fine frequency discrimination ability and can discriminate between the echolocation calls of different species (Schuchmann and Siemers 2010; Bastian and Jacobs 2015), different phonetic populations (Bastian and Jacobs 2015) and the sex of conspecifics (Schuchmann et al. 2012). Likewise, bats with frequency-modulated calls, as investigated here, might be capable of discriminating between very similar calls: *Myotis lucifugus* and *M. myotis* recognise individuals based on echolocation calls (Kazial et al. 2008; Yovel et al. 2009), female *Eptesicus fuscus* differentiate between sex (Kazial and Masters 2004) and *Noctilio albiventris* react with different suites of social behaviours that depend on individual familiarity and species identity (Voigt-Heucke et al. 2010). This might explain why our focal bats did not perceive *M. nattereri* playbacks as sufficiently similar in call design to show a positive reaction under the acoustic similarity hypothesis. The previously listed studies, however, were conducted in the lab, often repeating similar stimuli. While these studies show the discrimination ability of bats under ideal conditions, it remains unknown to what extent bats are also able to perform this discrimination while foraging under natural conditions in the wild. Only Knörnschild et al. 2012 tested call discrimination ability under natural conditions in the quasi-constant-frequency bat *Saccopteryx bilineata*, showing that males discriminate between sexes based on echolocation calls under natural and untrained conditions.

In addition to acoustic and foraging ecology similarity, other factors such as context, prey availability, competition or patch profitability will affect the attractiveness of acoustic simulations, particularly in the field. Firstly, information transfer between heterospecifics was intensively studied in the context of predator detection (McKee Shriner 1998; Vitousek et al. 2007; Magrath et al. 2009; Fallow and Magrath 2010; Fallow et al. 2011; Magrath and Bennett 2012; Getschow et al. 2013; Haff and Magrath 2013; Fuong et al. 2014), where it provides higher survival benefits and is likely more common than during foraging because (i) predation is a matter of life and death and (ii) predators often prey on multiple prey species. Reacting to heterospecific information in a foraging context may provide comparably fewer benefits due to non-identical foraging ecologies and entail higher costs due to competition. Secondly, prey availability in the field was uncontrolled and might have been sufficiently high not requiring the bats to attend and react to the calls of other individuals. Thirdly, the effect of playbacks in the field might be lower than in the flight room because calls of other close-by individuals in the field dilute the playbacks. Fourthly, our playbacks might not have simulated a sufficiently profitable food resource to warrant a reaction by the eavesdropping bats, despite simulating highly profitable foraging patches with prey capture rates at the top end of natural capture rates (12 captures per minute, 23–40 captures per minute of bat activity, and 100% capture rate = one capture per call sequence; Fig. S4). Previous studies observed up to 2–14 catching events per minute in vespertilionid bats (Griffin et al. 1960; Kalko 1995) with maximum capture rates of 1 capture every 2–3 s, and even up to 3 captures per second (Kalko 1995 and references therein). Average capture rates, however, are much lower: between 14 captures/min in pipistrelle bats (Kalko 1995), 2–10 mosquitoes per minute in *M. lucifugus* and *M. (subulatus) leibii* (Griffin et al. 1960), only 0.1–5 captures per minute in *Rhinopoma microphyllum* foraging on swarming ants (Cvikel et al. 2015) and 1.3 captures/min (quartiles 0–7.4) in *M. capaccinii/M. daubentonii* (this study). Thus, despite simulating one order of magnitude higher capture rates than occurring naturally at our experimental sites, we only found small effects. Interestingly, related playback studies which found positive phonotaxis towards playbacks presented mostly even higher capture rates, ranging from 24 to 30 feeding buzzes per minute of total time presented for 1 min (Übernickel et al. 2013) over 40 feeding buzzes/min presented over 3 min (Dechmann et al. 2009) and 60 feeding buzzes/min presented for 10 s (Dorado-Correa et al. 2013) to about 69 feeding buzzes/min for 12 s (Cvikel et al. 2015). Only one study demonstrated attraction of *Tadarida brasiliensis* towards only 6 feeding buzzes/min presented for 10 min (Gillam 2007). This suggests that bats are attracted to the playbacks of con- and heterospecific echolocation calls only when they simulate exceedingly profitable foraging patches. This raises the question if, to which extent and under which conditions bats naturally react to the calls of other individuals. It is possible that attraction increases gradually with feeding buzz rate, and was to date only detected experimentally when simulating highly profitable foraging patches. Lastly, experiments in the field are generally less controlled than in the flight room, making potential effects more difficult to detect due to additional noise.

However, even though the unexpected reaction in the flight room and the minor reaction in the field did not match our predictions, our study revealed a clear reaction towards *M. capaccinii* and *M. daubentonii* calls under certain conditions. We therefore show that conspecific and heterospecific information transfer takes place, but it appears to be context-dependent. Together with previous studies (e.g. Dorado-Correa et al. 2013; Übernickel et al. 2013), our study supports the general idea that interindividual information transfer based on echolocation calls is possible in bats, both within and across species boundaries. Both conspecific as well as heterospecific cues are thus used as a source of information by bats to guide their own decision making. Dorado-Correa et al. 2013 found heterospecific attraction between bats with partially overlapping foraging styles, yet could not exclude that the attraction was based on acoustic similarity between feeding buzz calls. Übernickel et al. 2013 found that two congeneric *Noctilio* species approached the acoustically similar feeding buzzes of each other. One of the species also approached the search calls of the other species, but not vice versa. Although search calls of those species differ by about 15 kHz in their initial constant-frequency part, the general call structure is similar between species and it remained unclear if the behavioural reaction was based on heterospecific recognition and similarity in foraging ecology, or on acoustic similarity. Likewise, our study does not support either of our hypotheses based on our initial similarity classifications. If bats were able to discriminate between the presented calls, our study suggests that bats recognise species identity, assess both the acoustic call structure and foraging ecology and react only if both match their own characters—which we can call the acoustic-and-foraging similarity hypothesis. On the contrary, if we assume that bats did not distinguish between calls of *M. capaccinii* and *M. daubentonii*, but those of *M. nattereri*, the acoustic similarity hypothesis would explain the observed behaviour more parsimoniously than the acoustic-and-foraging similarity hypothesis.

In summary, our study highlights the complex influences on information transfer within and between species. Further studies, both under controlled lab-conditions and in the field, where animal behaviour really happens, and in more species, are indispensable. These studies need to address the species-specific differences in information transfer and the influences of acoustic and (foraging) ecology similarity, social system, prey density and behavioural context.

## Electronic supplementary material

ESM 1 (PDF 1.27 mb)
