# Supplementary material for: Does similarity in call structure or foraging ecology explain interspecific information transfer in wild Myotis bats?
Source: Behav Ecol Sociobiol. 2017 Oct 29;71(11):168. doi: 10.1007/s00265-017-2398-x (PMC5661007; doi:10.1007/s00265-017-2398-x)
Supplement: Supplementary file 1 — (PDF 1.27 mb) [file 265_2017_2398_MOESM1_ESM.pdf]

Does similarity in call structure or foraging ecology explain interspecific information transfer in wild *Myotis* bats?

Theresa Hügel[1,2,3], Vincent van Meir[1,2], Amanda Muñoz-Meneses[2,4], B.-Markus Clarin[1], Björn M. Siemers[1,†], Holger R. Goerlitz[1,2,*]

[1] Sensory Ecology Group, Max Planck Institute for Ornithology, Seewiesen, Germany

[2] Acoustic and Functional Ecology Group, Max Planck Institute for Ornithology, Seewiesen, Germany

[3] Department of Animal Ecology and Tropical Biology (Zoology III), Julius Maximilian University of Würzburg, Würzburg, Germany

[4] Graduate School for Evolution, Ecology and Systematics, Ludwig Maximilian University of Munich, Planegg-Martinsried, Germany

* author for correspondence: hgoerlitz@orn.mpg.de

† deceased

SUPPLEMENTARY TABLES

Table S1: Duration of the echolocation call sequences used as playback stimuli in the experiments.

Bat species	Median (quartiles; min-max) duration (s)	N
M. capaccinii	**2.59** (2.32 – 2.69; 1.63 – 2.85)	55
M. daubentonii	**2.50** (2.19 – 2.85; 1.99 – 3.22)	23
P. pygmaeus	**2.23** (1.96 – 2.56; 1.25 – 2.84)	21
M. nattereri	**1.51** (1.34 – 1.94; 0.58 – 2.44)	17
N. leisleri	**2.26** (1.91 – 2.54; 0.97 – 2.77)	13

Table S2: Recording location, year, equipment and conditions for the call sequences used as playback stimuli in the experiments.

Bat species (number of sequences)	Location and year	Microphone	Microphone pre-amplifier	Sound card	Sampling frequency (kHz)
M. capaccinii (N = 55)	Bulgaria, 2011, water bodies	CM16, Avisoft	CMPA, Avisoft	USG 116 Hm, Avisoft	250
M. daubentonii (N = 23)	Germany, 2011, water bodies	CO-100K, Sanken	Ultralite-mk3, MOTU	Ultralite-mk3, MOTU	192
P. pygmaeus (N = 21)	England, 2011, hedgerows	CO-100K, Sanken	Quadmic, RME	USB-6251, National Instruments	250
M. nattereri (N = 17)	Germany, 2009, flight room	CM16, Avisoft	CMPA, Avisoft	USG 116 Hm, Avisoft	375
N. leisleri (N = 9)	Germany, 2011, open field	CM16, Avisoft	CMPA, Avisoft	USG 116 Hm, Avisoft	250
N. leisleri (N = 4)	England, 2009, open field	CO-100K, Sanken	Quadmic, RME	USB-6251, National Instruments	250

SUPPLEMENTARY FIGURES

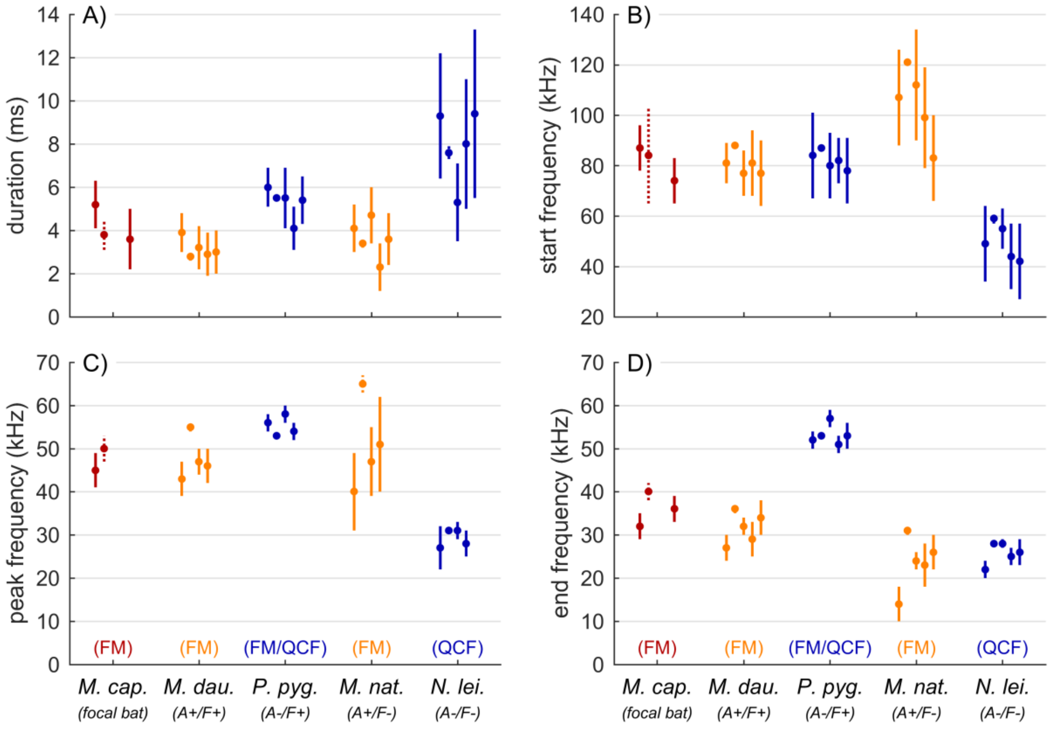

Fig. S1: Field-measured call parameters for the bat species used as playback stimuli. Data are literature values from field studies (see Table 1 for references) and are displayed as in these studies, either as mean ± std (solid lines) or mean ± sem (dotted line). Order of studies within species is as in Table 1 (from Ref. [1] to [5]). Species that we classified as acoustically similar or dissimilar to *M. capaccinii* (red) are shown in orange and blue, respectively, with a description of their general call shape (FM: frequency-modulated, QCF: quasi-constant-frequency). See Fig. 1 for exemplary call spectrograms of all species.

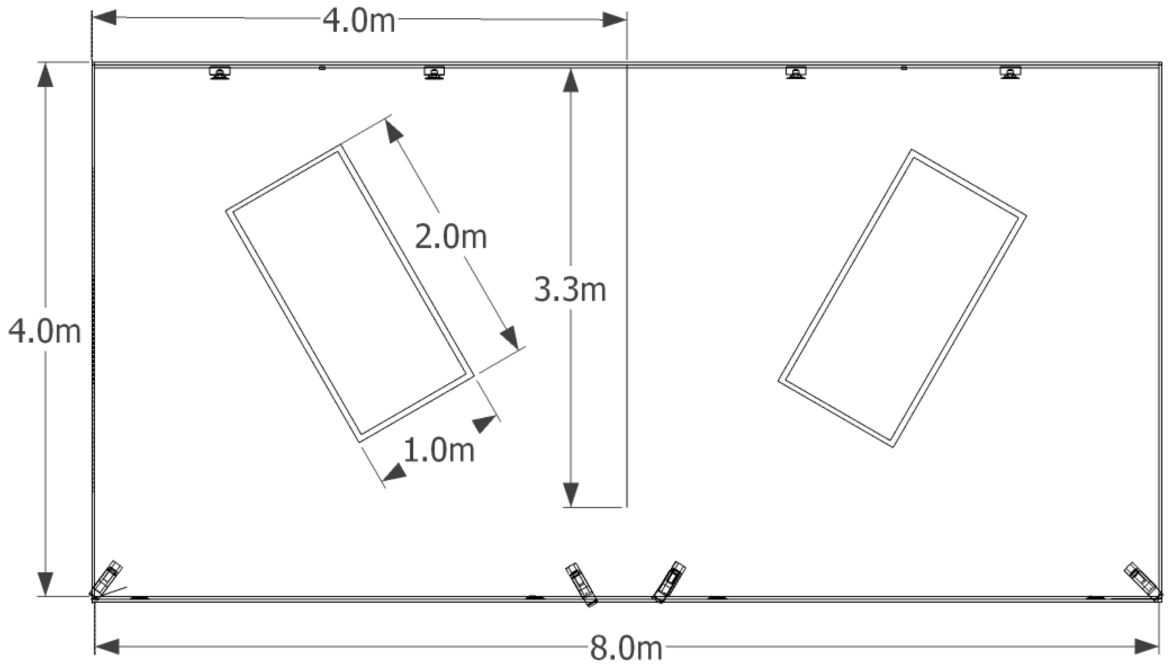

Fig. S2: Flight room of Experiment 1. The flight room was split into two compartments by a 3.3 m long curtain that left a 0.7 m wide opening at one side for the bats to pass between compartments. One water pool per compartment allowed the bats to forage. Two loudspeakers per compartment at the wall opposite to the opening presented echolocation call sequences of foraging bats. Four wide-angle video-cameras mounted in the corners monitored the behaviour of the bats in the complete flight room.

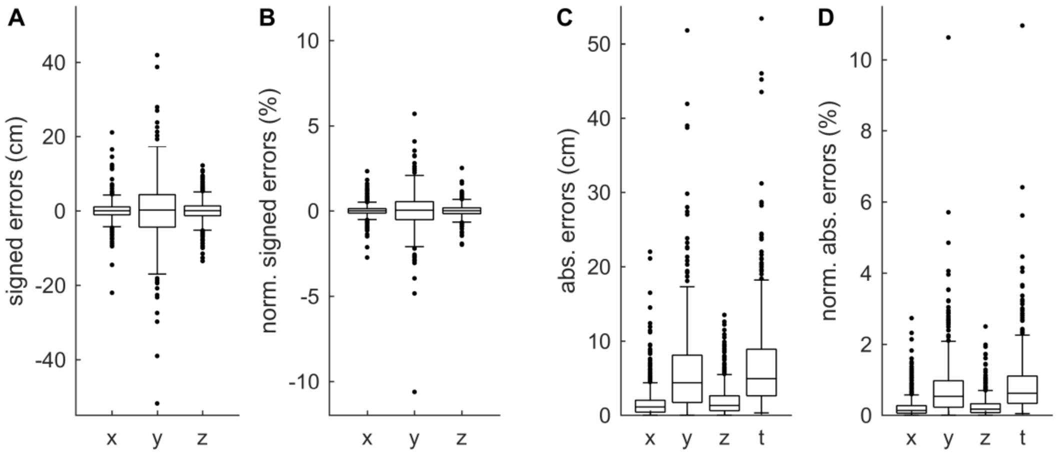

Fig. S3: Difference between measured and smoothed 3D bat positions.

A + B) Signed difference between the measured and the smoothed positions (measured – smoothed, **A**), and the signed difference normalized to the distance to the array (**B**), separately for the X-, Y- and Z-coordinates. Note that the signed differences are equally distributed around Zero, showing that the smoothing does not introduce a systematic bias and that the differences are rather due to random noise.

C + D) Absolute difference between the measured and the smoothed position (**C**), and the absolute error normalized to the distance to the array (**D**), separately for the X-, Y- and Z-coordinates and the total difference (labelled t) in 3D-space.

Differences of the Y-coordinate (direction away from the array) are larger than those of the X- and Z-coordinates, which is to be expected for 3D-triangulation methods. Note that median differences are still only around 5 cm for the Y-coordinate; and the upper quartile is still less than 10 cm.

Box plots present median, quartiles and whiskers at up to 1.5 times the interquartile range beyond the quartiles. N = 818 bat positions.

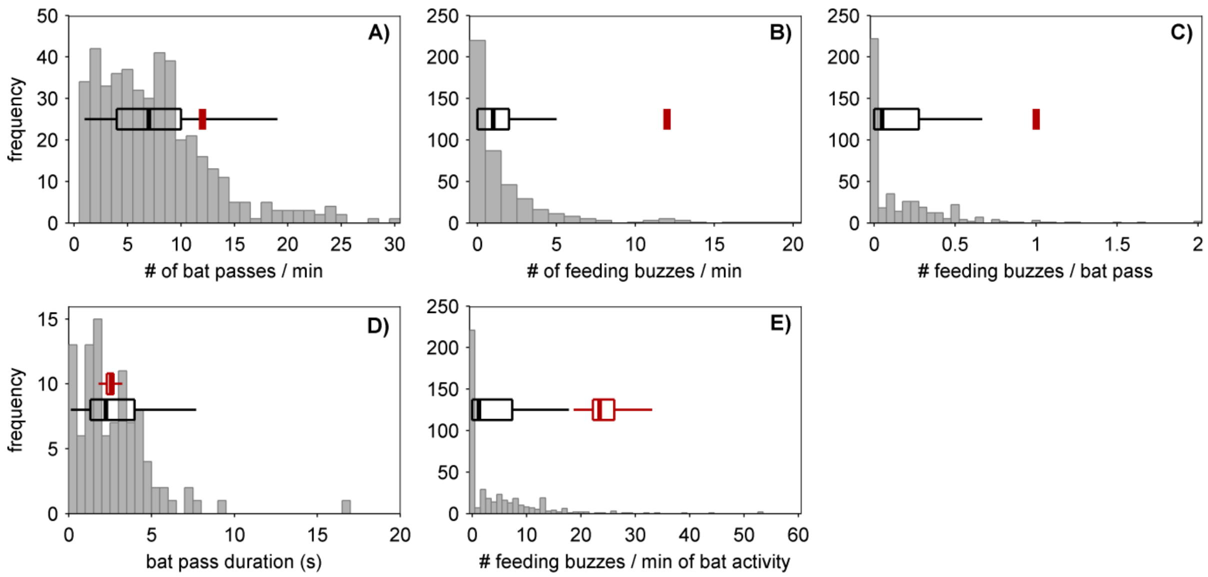

Fig. S4: Assessment of the playback stimuli as indicators of profitable foraging patches. Grey histograms and black box plots present data of 443 1-minute long pre-playback phases recorded in the field that contained at least one bat pass of *Myotis capaccinii/daubentonii*. Red box plots are the data for our 1-minute long playback stimuli for *Myotis capaccinii/daubentonii*. Box plots present median, quartiles and whiskers at up to 1.5 times the interquartile range beyond the quartiles.

A) The number of presented echolocation sequences (12/min) was roughly twice as high as the number of bat passes observed in the field (median: 7/min, quartiles: 4-10).

B) The number of presented feeding buzzes (12/min) was about 12 times higher than the number of feeding buzzes observed in the field (median: 1/min, quartiles: 0-2).

C) The presented capture rate of 1 feeding buzz per bat pass was about 20 times higher than the observed capture rate in the field (median: 0.05 feeding buzzes / bat pass, quartiles: 0-0.28).

D) The duration of presented echolocation sequences (median: 2.56 s, quartiles: 2.30-2.70) was equal to the duration recorded in the field (median: 2.26 s, quartiles: 1.32-3.97), although the variation in the field was larger than the presented variation. See Table S1 for the presented duration of all species.

E) The presented rate of feeding buzzes per minute of bat activity (median: 23.4 buzzes/min, quartiles: 22.18-26.12) is about 18 times higher than the rate observed in the field (1.3 buzzes/min, quartiles: 0-7.36).

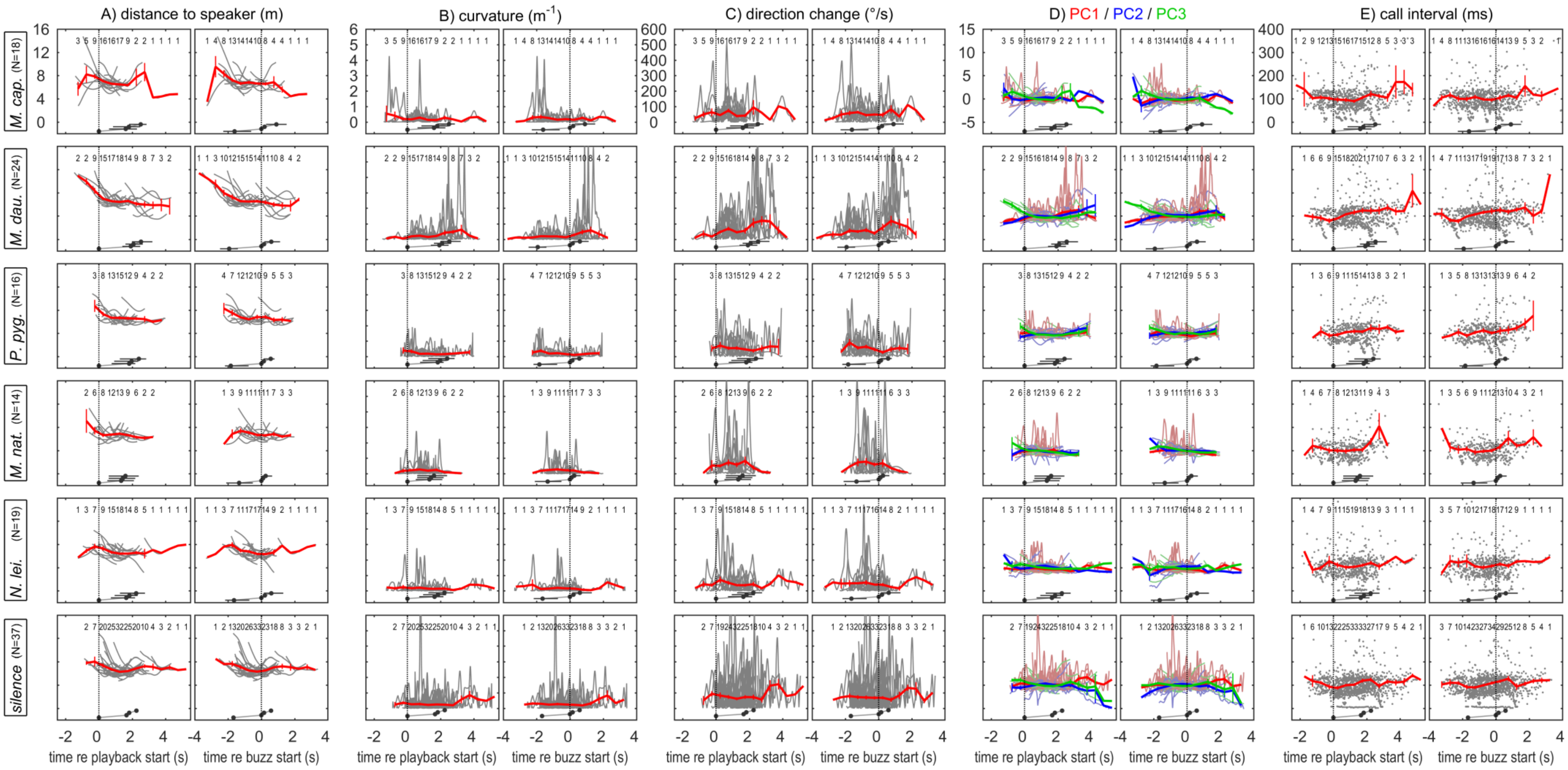

Fig. S5: Trajectory parameters, their PC-scores, and call intervals of *M. capaccinii* and *M. daubentonii* in the field.

Bat distance to the speaker (**A**), trajectory curvature (**B**), change in flight direction (**C**), the PC1-PC3 scores of the previous trajectory parameters and relative flight height (see Fig. 5D,E) (**D**) and call interval (**E**) as a function of playback species and time relative to the playback, with t=0

being the start of the playback (left panels) and start of the buzz (right panels). If bats approached the playbacks, we expected to see a reduced distance to the speaker and larger curvature and changes in flight direction. Lines are individual data (grey) and means ± SEM binned per 0.5 s (red). Small numbers at top indicate number of trajectories per bin. Dots with horizontal bars below the data indicate the mean playback time and the min/max-range across all playbacks for four time points within the playbacks: start of the whole playback, start of the feeding buzz, end of the feeding buzz, end of the whole playback.

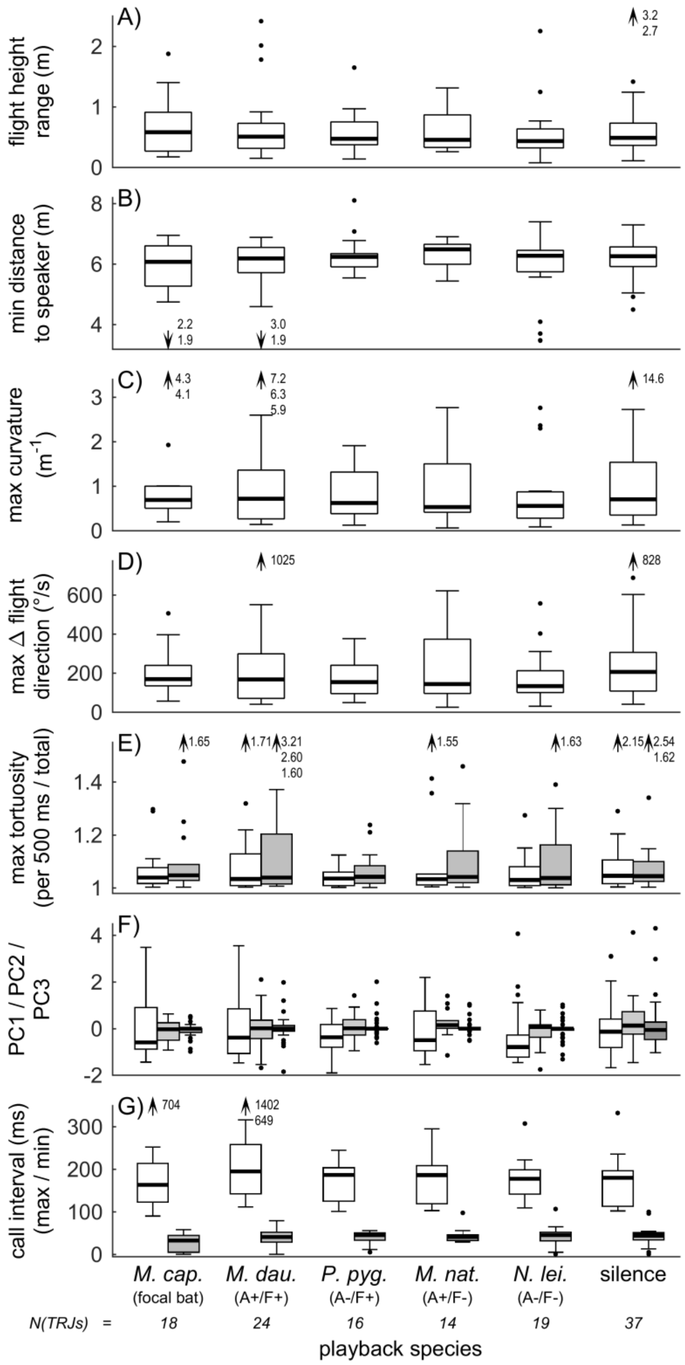

Fig. S6: Extreme values of flight and vocal behaviour per trajectory of *M. capaccinii* and *M. daubentonii* in the field. A) total range of flight height, B) minimum distance to loudspeaker, C-E) maximum values of trajectory parameters measuring trajectory curviness, F) PC1-PC3-scores of a PCA of the previous six flight trajectory parameters, G) minimum and maximum call interval. Box plots present median, quartiles and whiskers at up to 1.5 times the interquartile range beyond the quartiles. Extreme values beyond the axes limits are indicated next to the arrows.
